# Reconstruction of Bilateral Hindfoot Open Fracture Using the Femoral Head Allograft: A Three-Year Follow-Up Case Report

**DOI:** 10.7759/cureus.73964

**Published:** 2024-11-19

**Authors:** Amro Alhoukail, Salem Althuwaykh, Noura Alqahtani, Abdullah Alotaibi

**Affiliations:** 1 Orthopedic Surgery Department, King Fahad Medical City, Riyadh, SAU

**Keywords:** arthrodesis, bone loss, calcaneus defect, femoral head allograft, open calcaneus fracture, open talus fracture, reconstructive foot and ankle surgery

## Abstract

This case report presents a three-year follow-up of a young male with bilateral hindfoot fractures due to a high-energy road traffic accident, resulting in a comminuted open calcaneal fracture on the left and an open fracture-dislocation of the right talus. Staged reconstruction was performed, including initial debridement, temporary cement spacers, and subsequent fixation with femoral head allografts (FHAs). The right foot underwent a tibiotalocalcaneal (TTC) fusion, and the left foot received a double arthrodesis. Follow-up imaging confirmed allograft integration and complete fusion. At three years, the patient had a stable, plantigrade foot and satisfactory function for daily activities. This case demonstrates the successful use of FHAs for complex hindfoot reconstructions, preserving limb length and functionality.

## Introduction

Foot fractures and dislocations exhibit a diverse range of patterns, influenced by the foot's position at the time of injury as well as the magnitude and direction of the applied force. High-energy injuries to the hindfoot, such as those resulting from road traffic accidents, often result in complex trauma that is associated with poorer outcomes and a high risk of significant long-term disability [1]. Open talar and calcaneal injuries of the hindfoot are a formidable orthopedic challenge. The soft-tissue disruption associated with these high-energy traumatic injuries adds to the complexity of treatment [2,3].

Significant osseous defects in the hindfoot and ankle present a challenging scenario for surgical intervention. Tibiotalocalcaneal (TTC) arthrodesis with a structural allograft is often necessary to address the bone void, maintain limb length, and achieve successful fusion [4].

Limb shortening resulting from structural bone loss in TTC arthrodesis poses a significant concern, as it can adversely affect a patient’s gait and weight-bearing capacity. To mitigate this risk, femoral head allografts (FHAs) have proven effective in preserving limb length in patients with substantial bone deficits [5].

Numerous studies in the literature discuss the outcomes of using FHAs for talar bone defects; however, there is a paucity of evidence regarding their application in calcaneal bone defects. This case report presents a challenging scenario of a bilateral hindfoot open fracture with substantial bone loss of left calcaneus and right talus in a young male, managed through staged reconstructive surgeries with FHAs.

## Case presentation

**Figure 1:**
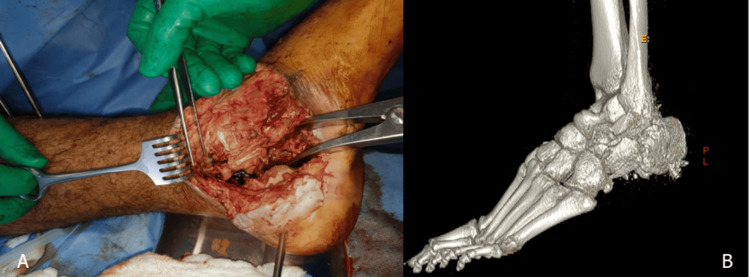
(A) Intraoperative and (B) 3D CT image of medial aspect of the left foot showing calcaneal defect.

**Figure 2:**
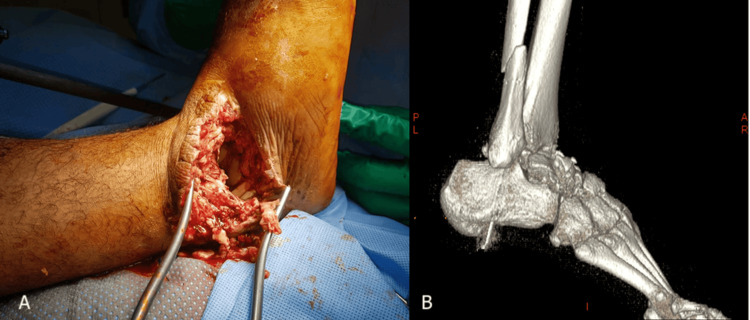
(A) Intraoperative and (B) 3D CT image of the lateral aspect of right foot with intraoperative image of fracture dislocated right talus.

A 35-year-old otherwise healthy male sustained polytrauma, including a highly comminuted open left calcaneal fracture (Figure 1) and an open fracture dislocation of the right talus (Figure 2) after a road traffic accident in September 2019. Initially managed in a local hospital, he was subsequently transferred to our tertiary care center for further treatment.

**Figure 3:**
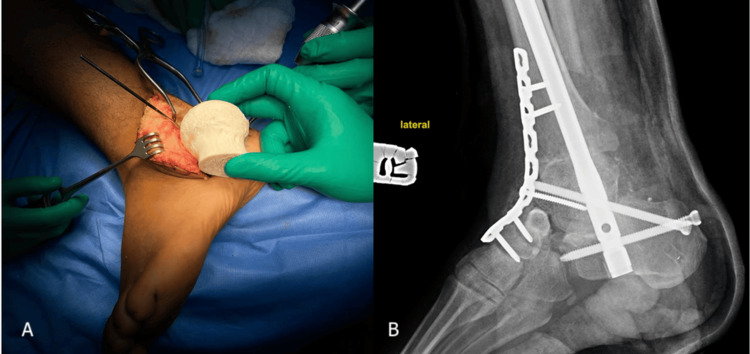
(A) Intraoperative and (B) X-ray image of the right foot showing femoral head allograft for talus defect reconstruction with tibiotalocalcaneal (TTC) fusion using a retrograde TTC nail with 3.5 LCD plate.

**Figure 4:**
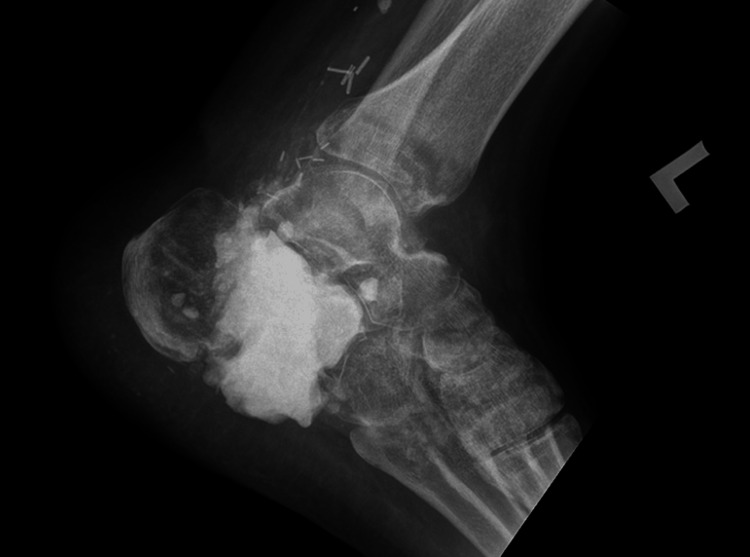
The left foot’s comminuted calcaneal fracture was managed initially with debridement and filling of the osseous void with an antibiotic-impregnated bone cement spacer.

In our center, bilateral foot open fractures were treated in stages that involved multiple debridement of the wounds. Right open talus fracture-dislocation was finally reconstructed by FHA and TTC fusion using a retrograde TTC nail with a 3.5 LCD plate spanning from the anterior ankle to the midfoot (Figure 3). The left foot’s comminuted calcaneal fracture was managed initially with debridement and filling of the osseous void with an antibiotic-impregnated bone cement spacer (Figure 4), followed by soft tissue coverage using a free flap.

**Figure 5:**
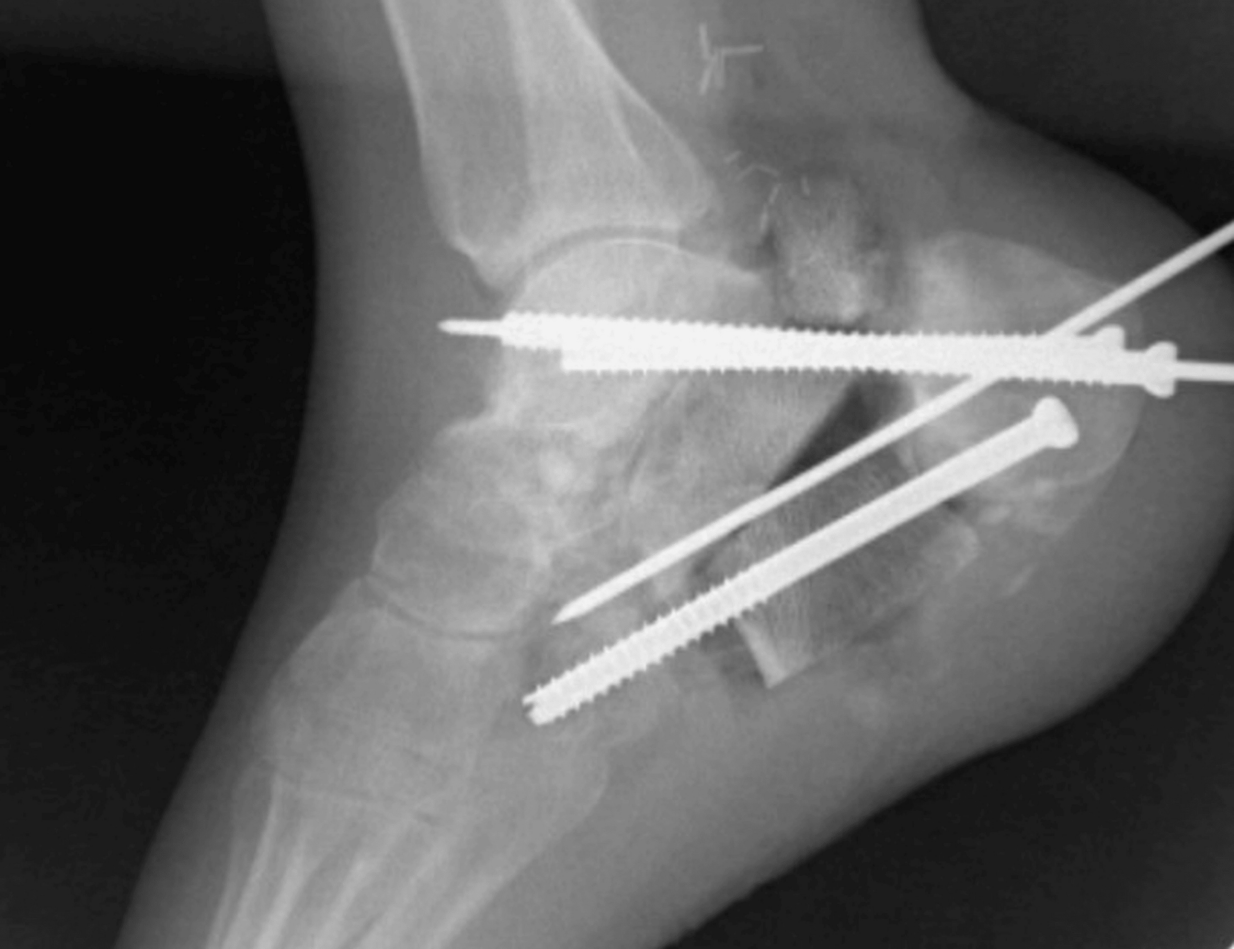
Intraoperative X-ray image showing a large calcaneus bone defect filled with a structural femoral head allograft and arthrodesis of the subtalar and calcaneocuboid joints.

Approximately one year later, after flap integration and successful weight-bearing, a second-stage procedure was performed using an extensile calcaneal approach. The bone cement spacer was removed, and additional debridement was conducted. Measurements were taken to ensure appropriate allograft placement, and the remaining space between the calcaneal tuberosity, talus, and cuboid was filled with an FHA. A double fusion with the talus and cuboid was then secured using three 6.5 mm cancellous cannulated screws under fluoroscopic guidance (Figure 5). The surgical site was closed, and a cast was applied.

**Figure 6:**
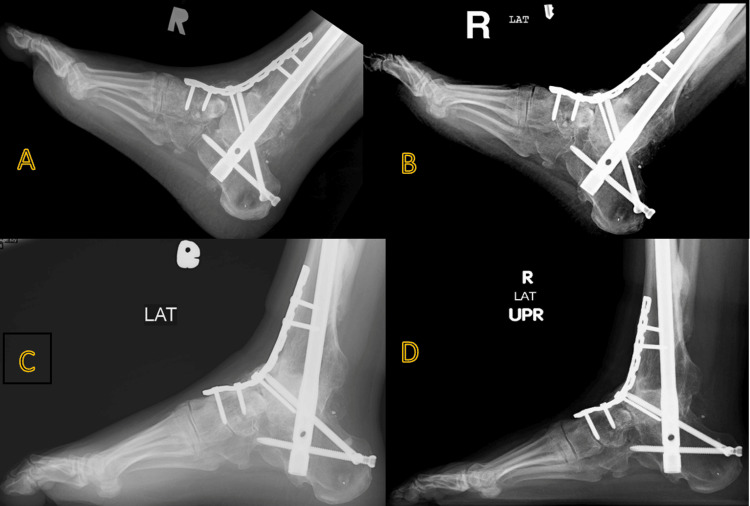
Right lateral foot X-ray (A) Two months post-procedure; (B) One year post-procedure; (C) Two years post-procedure; (D) Three years post-procedure

**Figure 7:**
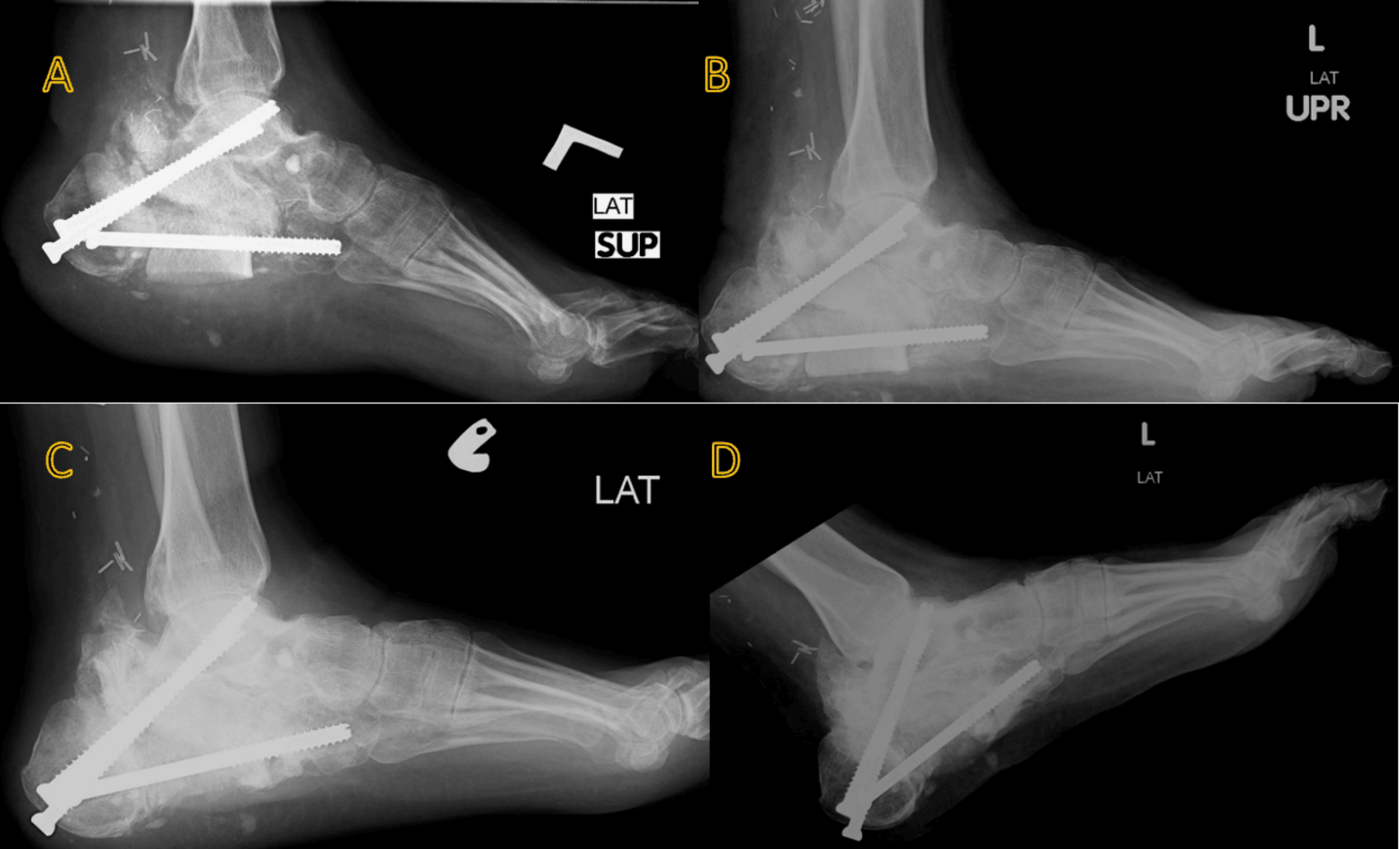
Left lateral foot X-ray (A) Two months post-procedure; (B) Six months post-procedure; (C) One year post-procedure; (D) Eighteen months post-procedure

The patient was followed up in the clinic with serial X-rays for three years after surgery (Figures 6-7). At three months postoperative, a CT scan revealed complete union and incorporation of the FHA with native bone. In the postoperative protocol, the patient was kept non-weight bearing until eight weeks after surgery, then he was allowed to bear weight as tolerated using an air cast. During his long follow-up, he reported no significant discomfort. The surgical wounds have completely healed. Now he has bilateral, stable plantigrade feet with satisfactory functional ability in daily activities. He was involved in an extensive rehabilitation program.

## Discussion

Hindfoot bone defects of significant size can be successfully treated using autografts or allografts. However, the utilization of autologous bone grafts is restricted due to limited sources and the morbidity associated with donor sites, rendering it a less favorable choice for treating extensive bone defects. On the other hand, allogeneic bone grafts offer a readily available solution for filling large bone defects; yet, there is a risk of potential collapse if they fail to integrate properly with the recipient's native bone tissue [3,6].

To address significant bone loss, bulk structural allografts are commonly used to reconstruct the talar/calcaneus defect and restore limb length. Over the past two decades, FHAs have been utilized to compensate for the gap left by large bone defects. Complication rates ranging from 46% to 86% have been documented [7-9]. Recent studies have shown that the rates for bulk FHA fusion are greater than previously documented [10-12].

Tibiocalcaneal fusion has been documented as efficacious in achieving fusion, although a majority of patients experienced a notable reduction in length [13-16]. Blair described the utilization of a sliding tibial graft in TTC fusion [17]. Although limb length and movement at the talonavicular joint can be preserved with a normal ankle contour, an unfixed graft positioned anterior to the weight-bearing axis is intrinsically unstable and may result in nonunion [14,16].

Lee et al. reported the results of their TTC fusion study in 34 patients who were treated with a retrograde nail. The patients were followed up for a minimum of two years. Out of the 34 patients, fusion was successfully achieved in 28 individuals, accounting for 82% of the cases. However, the authors discovered that having uncontrolled diabetes increases the risk of nonunion [18].

Coetzee with his colleagues published their ankle and TTC fusion results using FHA in 45 patients with a mean follow-up of 42.8. Fusion rate was achieved in 90% of both the ankle and TTC fusions. The use of these grafts markedly enhances patients' pain relief and functionality [16]. In a different study of 32 patients who had TTC arthrodesis with a bulk FHA, the radiographic fusion rate was only 50%, and diabetes mellitus was found to be the only notable risk factor for nonunion [19].

## Conclusions

In conclusion, we present the use of FHAs for the reconstruction of bilateral hindfoot bone loss, demonstrating an effective approach for hindfoot reconstruction in complex clinical settings. Long-term follow-up of our case revealed complete fusion, satisfactory functional abilities in daily activities, and no significant discomfort. This case emphasizes the potential benefits of FHAs in preserving limb length and restoring function in patients with substantial bone deficits following high-energy trauma. Additionally, it highlights the importance of a staged reconstructive approach, which allows for both soft tissue healing and optimal graft incorporation, ultimately contributing to successful long-term outcomes.
